# Awareness Level About Lead Poisoning Among the Saudi Population in Arar City, Saudi Arabia: A Cross-Sectional Study

**DOI:** 10.7759/cureus.62112

**Published:** 2024-06-10

**Authors:** Ekramy Elmorsy, Rawwabi Satam R Alshammari, Rashed Satam B Alshammari, Rasha Mohammed M Alanazi, Nirah Mohammed M Alanazi

**Affiliations:** 1 Pathology, Northern Border University, Arar, SAU; 2 College of Medicine, Northern Border University, Arar, SAU

**Keywords:** lead toxicity, neurodevelopmental disorders, awareness, lead, heavy metal toxicology

## Abstract

Objectives: This study aimed to evaluate the awareness regarding lead poisoning among the Saudi population of Arar, Saudi Arabia.

Methods: A cross-sectional, online self-administered questionnaire-based method was utilized for data collection through convenience sampling from Arar, Saudi Arabia. The collected data were analyzed using descriptive and inferential statistics.

Results: The study included 389 participants, with a majority (302 participants (77.6%)) of females with a mean age of 31.7 years (range 18-54 years). The source of information about lead poisoning was reported as the media in 264 participants (67.8%). The participants (197, 50.6%) reported children as the age group most at risk for lead poisoning. All suggested sources of exposure to lead were identified by 190 participants (48.9%). As for the most common effect of lead poisoning, mental impairment was reported by 101 participants (26%), while 107 (27.6%) participants reported nerve problems. Kidney problems were reported by 181 participants (46.4%). Gender significantly (P = 0.001) affects the participants' knowledge about the sources of lead poisoning, while knowledge about the routes of poisoning was significantly affected by the participants’ age (P = 0.010).

Conclusion: In Saudi Arabia, there is limited knowledge of risk factors, route of poisoning, and management and prevention of lead poisoning. A comprehensive lead-poisoning prevention strategy must include a high level of public knowledge of lead hazards as a key component.

## Introduction

Lead (Pb) is a widely occurring heavy metal, which is present in small amounts in the soil, water, air, and even inside our homes [1]. Because it is widely used, simple to extract, and simple to work with, poisoning can happen with it frequently. Although it has some useful applications, it can also be toxic to people and animals, which can have dangerous consequences [2]. Its broad use has ceased in many nations, but it is still utilized in a variety of industries, including auto repair, battery production and recycling, refining, smelting, and other processes. Nearly every organ in the body is affected by the extremely toxic element lead [3]. 

Lead poisoning mostly happens when tainted food or water is consumed. Lead is thought to enter the bloodstream swiftly and is known to negatively impact several organ systems, including the immune system, kidneys, central nervous system, and cardiovascular system [1]. The majority of pharmaceutical firms have established a daily maximum lead consumption of 1.0 μg/g; nonetheless, extended exposure to even this low level of lead can be harmful to humans. Blood lead levels are also raised as a result of occupational exposure. Girls who have higher blood levels tend to go through puberty later [2-3]. The blood lead levels that can be considered as safe without health hazards have not been identified yet. Children's cognitive capacity was discovered to be reduced by extremely low but permanent levels of lead exposure [1,4].

Lead toxicities (acute or chronic) can result in kidney, brain toxicities, hypertension, and anemia. Lead was reported to cause seizures and even death at very high exposure levels. Even at low doses, lead exposure can have serious long-term effects on a child's behavior and neurological development including retarded growth, children's antisocial behavior, a shorter attention span, and delayed learning, due to alteration in the absorption of minerals required for the growth and action of brain and nerves [5]. Chronic exposure to Pb can cause sadness, numbness, and tingling in the extremities, nausea, abdominal pain, loss of coordination, and short-term memory or attention problems. Chronic lead poisoning also causes anemia, drowsiness, headaches, fatigue, slurred speech, and stupor [6].

Elevated blood lead levels during pregnancy increase the risk of low birth weight or early birth. Even with blood lead concentrations significantly lower than 25 μg per deciliter, the fetus may suffer negative consequences [1]. Blood lead levels in neonates were found to be higher than simultaneous maternal lead levels. Children are more affected by Pb exposure as they are more exposed to dust or dirt on their hands, toys, or other items that may contain lead, and their bodies absorb more Pb than those of adults with reported higher rates of Pb absorption than adults [7,8].

To detect an elevated lead level in the body, whole blood lead level tests are used [9]. The most important nutrients for reducing lead absorption seem to be vitamin C, calcium, iron, and, to a lesser extent, zinc and phosphorus. Moreover, vitamin D and vitamin B9 (folate) can reduce the blood lead level. In addition, thiamine, a vitamin in group B, increases brain lead excretion [10].

Future initiatives to maintain reductions in lead exposure at various levels should combine comparable household- and individual-level awareness-raising efforts with regulatory action and longer-term initiatives. The current proposed study aims to evaluate the awareness of the public regarding Pb poisoning according to the routes of exposure and the common toxic effects among the general population of Arar, Saudi Arabia.

## Materials and methods

Study design

This was a cross-sectional study conducted between September 2023 and February 2024 to assess the awareness of the public regarding lead poisoning among the Saudi population aged above 18 years in Arar, Saudi Arabia. Responses from Non-Saudi participants or Saudi nationals aged below 18 years were excluded in the final collected data analysis.

Data were collected using the adapted questionnaire from Sarfaraz et al. [8] with some modifications. The translated version of the questionnaire was validated via distribution to 20 volunteers to check that the translation was clear, and the questions were easily self-administered. The responses for validation were not included in the final data analysis. The questionnaire is composed of three sections. The first section covered the subjects' sociodemographic characteristics (age, gender, marital status, residence, and educational level). The second section covered lead poisoning-related information including the type of population mostly affected, sources, the common effects, and the treatment. The third section was composed of one question about the source of knowledge of the participants regarding lead poisoning.

The questionnaire was prepared in an online version, which was distributed to the public population through the specific groups of social media platforms covering the different groups of the Saudi population in Arar, comprising Facebook and Twitter, and included questions that meet the study objectives.

The sample size was identified utilizing the equation n = z2p (1-p)/e2 (n = sample size, z = degree of confidence based on the standard normal distribution, p = approximate proportion of the population that exhibits the trait, and e = tolerated margin of error). A proper sampling method was utilized. The sample for robust data is expected to be more than 384 participants from various age groups, occupations, and educational levels.

Ethical considerations

Ethical approval to conduct the study was obtained from the local bioethics committee of Northern Border University, Arar, Saudi Arabia (decision number 70/44/H). The questionnaire proceeded with a brief explanation of its purpose and aims and a reminder that participation is completely voluntary. All responses were kept secure and confidential through all phases of the research project.

Statistical analysis

The data were analyzed using SPSS version 26 (Statistical Package for Social Sciences) (IBM Corp., Armonk, NY). Cronbach's alpha test was used to check for the internal consistency of the participants' responses. Descriptive statistics was used for the prevalence and quantitative variables. We evaluated the categorical risk factors using the X^2^ test. Statistical significance was determined by a p-value lower than 0.05.

## Results

The current study was conducted to evaluate the levels of awareness about lead poisoning among Saudi population in Arar, Saudi Arabia, and to evaluate the effect of the different demographic data on the different areas of awareness about lead poisoning. The study included 389 participants; 77.6% (302 participants) of them were females, while 22.4%(87 participants) were males with a mean age of 31.7 years (range 18-54 years). As for education, 76.2% of the participants were university-educated or more (Table 1). The participants’ response internal consistency was evaluated by Cronbach's alpha test. Cronbach’s alpha estimated value was 0.89, which indicated strong consistency of the participants' responses to survey questions prior to further data analysis.

**Table 1:** Sociodemographic characteristics of the participants (n = 389)

Parameter		No.	Percent
Gender	Male	87	22.4
	Female	302	77.6
Age	Less than 20	68	17.5
	21-30	147	37.7
	31-40	75	19.4
	41-50	89	23.0
	50-60	10	2.5
Educational status	Illiterate	3	0.8
	Elementary	2	0.5
	Middle	11	2.7
	Secondary	77	19.7
	University or more	297	76.2

Data regarding the participants’ responses to the knowledge section questions are shown in Table 2. Responses show that 50.5% of the participants reported children as the age group most at risk for lead poisoning. Regarding the source of lead poisoning, 190 participants (46.8%) were aware of all included choices as potential sources of exposure to Pb, including makeup, plumping tools, children’s toys, pencils, and air pollution. As for the most common effect of lead poisoning, renal impairment was the most commonly known effect of Pd toxicity among the participants (reported by 46.4%), followed by nerve problems (27.6%) and mental impairment (26%). About the sources of exposure to lead, 54.6% of the participants were aware of all sources of exposure, including inhalation, ingestion, and skin contamination. The effect of lead on intrauterine unborn children was known by 83.3% of the participants. Interestingly, 51.9% of the participants reported that iron, calcium, and vitamin C deficiencies indicate lead poisoning, while only 20.2% of the participants were aware of the treatment for lead poisoning.

**Table 2:** Participants' knowledge of risk factors, effects, management, and prevention of lead poisoning (n = 389)

Parameter		No.	%
Age group at risk exposed to lead poisoning	Children	197	50.6
	Adults	95	24.3
	Teens	70	18.0
	Elderly	28	7.1
Sources of lead poisoning	Plumbing tools	30	7.7
	Pencils	29	7.4
	Children toys	30	7.7
	Pots	14	3.6
	Soil	4	1.1
	Plants	10	2.5
	Makeup	44	11.2
	Air pollution	20	5.2
	All of the above	190	48.9
	None of the above	19	4.9
Most common effect of lead poisoning	Mental impairment	101	26.0
	Nerve problems	107	27.6
	Kidney problems	181	46.5
Most common ways to get lead poisoning	Inhale	29	7.4
	Inhalation and mouth	43	10.9
	Skin	49	12.6
	Oral	56	14.5
	All the above	213	54.7
Lead affect unborn children	Yes	324	83.3
	No	65	16.7
Lead affect fetuses	Diseases in the long term	46	11.8
	Fall of the fetus	15	3.8
	Birth defects	86	22.1
	Premature birth	18	4.6
	Nll the above	224	57.7
Iron, Calcium and Vitamin C Deficiencies Indicate Lead Poisoning	Yes	202	51.9
	No	187	48.1
Know the treatment for lead poisoning	Yes	79	20.2
	No	310	79.8
Preventive measure to overcome lead poisoning	Healthy food	21	5.5
	Reduce exposure to lead-containing materials	164	42.1
	Raising awareness	185	47.5
	Cleaning hands	19	4.9

The effects of age, gender, and educational levels on the participants are shown in Table 3. There was a significant association between the participants’ age and education with their knowledge of the age group at risk for lead poisoning (P= 0.007 and 0.026, respectively). Knowledge of the source of lead poisoning was significant with the participants' gender (P = 0.001). Knowledge of the route of poisoning was significant with the participants’ age (P = 0.010). Knowledge of the treatment for lead poisoning was associated with age (P = 0.029). Knowledge of preventive measures to overcome lead poisoning was associated with age and education (P = 0.047 and 0.001, respectively).

**Table 3:** Participants' knowledge of risk factors, effects, management, and prevention of lead poisoning in association with age, gender, and education of the participants (n = 389). * means p-value <0.05, ** means p-value <0.01.

Parameter		No.	%	Association with age (P-value)	Association with gender (P-value) *	Association with education (P-value)
Age group at risk exposed to lead poisoning.	Children	197	50.6	0.007**	0.896	0.026*
	Adults	95	24.3			
	Teens	70	18.0			
	Elderly	28	7.1			
Sources of lead poisoning	Plumbing tools	30	7.7	0.282	0.001**	0.130
	Pencils	29	7.4			
	Games	30	7.7			
	Pots	14	3.6			
	Soil	4	1.1			
	Plants	10	2.5			
	Makeup	44	11.2			
	Air pollution	20	5.2			
	All the above	190	48.9			
	None of the above	19	4.9			
Most common effect of lead poisoning	Mental impairment	101	26.0	0.126	0.082	0.094
	Nerve problems	107	27.6			
	Kidney problems	181	46.5			
Most common ways to get lead poisoning	Inhalation	29	7.4	0.010*	0.053	0.063
	Inhalation and mouth	43	10.9			
	Skin	49	12.6			
	Oral	56	14.5			
	All of the above	213	54.7			
Lead effect on unborn children	Yes	324	83.3	0.088	0.033*	0.279
	No	65	16.7			
Lead effect on fetuses	Diseases in the long term	46	11.8	0.353	0.707	0.001**
	Fall of the fetus	15	3.8			
	Birth defects	86	22.1			
	Premature birth	18	4.6			
	All of the above	224	57.7			
Iron, calcium, and vitamin C deficiencies indicate lead poisoning.	Yes	202	51.9	0.565	0.034*	0.698
	No	187	48.1			
Know the treatment for lead poisoning	Yes	79	20.2	0.029*	0.421	0.393
	No	310	79.8			
Preventive measures to overcome lead poisoning	Healthy food	21	5.5	0.047*	0.915	0.001**
	Reduce exposure to lead-containing materials	164	42.1			
	Raising awareness	185	47.5			
	Cleaning hands	19	4.9			

According to the participant's responses, the main source of information about lead poisoning was the media among 67.8% of the participants and through education curriculum and lead poisoning workshops, reported by 26.2% and 6% of the participants, respectively.

## Discussion

Most people think of lead as an environmental pollutant. Food, water, soil, and air are the main routes via which it enters the human body. In addition, adults are exposed to lead at work, which causes them to "bring lead home" with their clothing. Although lead was previously a metal found in the crust of the earth, mining and other uses made it a risk to human health. Efforts to avoid lead poisoning in children are hampered by low levels of awareness, whereas high levels of knowledge have been shown to be crucial in lowering childhood lead exposure. Thus, lead hazard awareness campaigns are a crucial cornerstone of successful lead exposure control strategies [11].

This study has demonstrated that, even in places where lead exposure risk has already been identified, there is widespread lead pollution and little awareness of the problems associated with it. This was in line with a study in South Africa, which reported similar results [12]. The study reported that only 11% of the study participants had ever heard of lead; most associated it with lead in petrol [12].

More than 90% of kids in Cape Province, South Africa's urban and rural populations, have blood lead levels below 10 mg/dl, according to recent prevalence studies. Children's exposure to lead may be a significant urban health issue across Africa, according to studies conducted in other nations [13,14]. In our study, 39.6% of the participants were exposed to lead poison before. Economic benefits are significant if childhood lead exposure is reduced, perhaps even more so than has been reported from wealthy nations [13,15].

Ingestion and inhalation of dust can expose children to lead [13]. Because they frequently put their hands in their mouths and spend a lot of time in areas where lead particles from degraded paint, petrol fumes, and smelting activities are deposited, infants and young children are at a particularly high risk of exposure. Ingestion of water delivered through lead plumbing, inhalation of pollutants from automobiles using leaded fuel, and eating food stored in lead-soldered cans or lead-glazed ceramics are a few additional common forms of exposure. Children may be at risk from dozens of other sources of lead, depending on where they live, their family's cultural traditions, their line of work, and their interests [13,16]. In our study, the source of lead poisoning was reported as 12.2% makeup, 7.7% plumping tools, 7.7% children’s toys, 7.4% pencils, and 5% air pollution. The most common ways to get lead poisoning were reported as inhalation only (7.4%), inhalation and mouth (10.9%), skin (12.6%), and oral ingestion (14.5%).

Convulsions, coma, and even death can occur as a result of extremely high lead exposures, which raise a child's blood lead level to roughly 100 micrograms per deciliter (g/dL) of whole blood. Early studies that tried to show a link between low-level exposure and children's cognitive function used cross-sectional research designs, which evaluate blood lead levels and IQ test scores simultaneously, usually after age five or six [17]. Additional measures are typically taken to quantify the influence of other factors that affect cognitive development and that can muddle the relationship between children's intelligence and their blood lead concentration because there are a variety of genetic and environmental factors that influence cognitive development, independent of lead exposure [18].

This report outlined the awareness about lead exposure in Arar, Saudi Arabia. In Saudi Arabia, the minimum limit for lead content in the home environment is enforced either very little or not at all. The health effects of these exposures are not widely known, and other health issues and infectious disorders are competing for attention. The present study was carried out through an online survey, which offered the following benefits: affordability, elevated response rates, and ease of use. However, the data are subject to the same constraints as online survey-based research, including sampling bias, respondent access concerns, and fabricated answers in biased samples. Meanwhile, Internet surveys are a commonly used and quickly growing technique for gathering data from cross-sectional surveys.

## Conclusions

In Saudi Arabia, there is limited knowledge of risk factors, route of poisoning, management, and prevention of lead poisoning. The cost of lead hazard awareness projects is generally modest, and they have been shown to be beneficial, even if some of the treatments needed to lower the risk of childhood lead exposure may be expensive and difficult. A comprehensive lead-poisoning prevention strategy must include a high level of public knowledge of lead hazards as a key component. It is possible to close this significant knowledge gap in public health and reduce the risk of lead poisoning in young children and unborn children through programs to increase awareness of lead dangers, particularly among parents and pregnant women. These educational programs ought to concentrate on regional lead sources, including paint, lead used in cottage industries, para-occupational lead exposure, and lead-related hobbies.
